# The Biological Function and Clinical Utilization of CD147 in Human Diseases: A Review of the Current Scientific Literature

**DOI:** 10.3390/ijms151017411

**Published:** 2014-09-29

**Authors:** Lijuan Xiong, Carl K. Edwards, Lijun Zhou

**Affiliations:** 1 Central Laboratory, Navy General Hospital, Beijing 100048, China; E-Mail: xljxyh2008@163.com; 2 National Key Laboratory of Biotherapy and Cancer Research (NKLB), West China Hospital and Medical School, Sichuan University, Chengdu 610041, China; E-Mail: drcarledwards@gmail.com; 3 Department of Dermatology, University of Colorado at Denver, Anschutz Medical Center, Aurora, CO 80025, USA

**Keywords:** CD147, EMMPRIN, biological function, pathogenesis, diagnostic biomarkers, therapeutic target, matrix metalloproteinases (MMPs)

## Abstract

CD147 or EMMPRIN is a member of the immunoglobulin superfamily in humans. It is widely expressed in human tumors and plays a central role in the progression of many cancers by stimulating the secretion of matrix metalloproteinases (MMPs) and cytokines. CD147 regulates cell proliferation, apoptosis, and tumor cell migration, metastasis and differentiation, especially under hypoxic conditions. CD147 is also important to many organ systems. This review will provide a detailed overview of the discovery, characterization, molecular structure, diverse biological functions and regulatory mechanisms of CD147 in human physiological and pathological processes. In particular, recent studies have demonstrated the potential application of CD147 not only as a phenotypic marker of activated regulatory T cells but also as a potential diagnostic marker for early-stage disease. Moreover, CD147 is recognized as an effective therapeutic target for hepatocellular carcinoma (HCC) and other cancers, and exciting clinical progress has been made in HCC treatment using CD147-directed monoclonal antibodies.

## 1. Introduction

Cluster of differentiation 147 (CD147) or extracellular matrix metalloproteinase inducer (EMMPRIN) is a transmembrane glycoprotein, also known as basigin (BSG), that is encoded by the *basigin* gene [1,2,3]. CD147 is a member of the immunoglobulin superfamily with a structure related to the putative primordial form observed in this family [3,4] and plays a role in intercellular recognition [5]. As a type I integral membrane receptor, CD147 has many ligands, such as cyclophilin proteins, *Plasmodium falciparum* reticulocyte binding-like homologue 5 (PfRh5), and integrins.

CD147 is expressed in many tissues and cells. Over the past 5 years, several groups have shown that CD147 acts as a key molecule in the pathogenesis of several human diseases. For instance, CD147 is an obligatory assembly factor for monocarboxylate transporters (MCTs) [6], which play roles in various pathological processes. Indeed, CD147 possesses a diverse range of functions in human healthy tissues and diseases, especially cancers. It is important to characterize the molecular events in cancers in detail. An emerging widespread hallmark of cancer is altered energy metabolism, *i.e.*, aerobic glycolysis (Warburg effect), as first observed by Otto Warburg [7,8]. In particular, cancer cells exhibit elevated rates of glucose consumption and high lactate production in the presence of oxygen. Increasing strength of the Warburg effect drives both tumor growth and the spread of metastases and is associated with poor outcomes in cancers [8]. Under hypoxic conditions, CD147 promotes tumor growth, inhibits tumor cell apoptosis and enhances the invasion ability of malignant tumors, thus, CD147 may contribute to the Warburg effect. Under a hypoxic microenvironment, malignant tumor cells rapidly reproduce, using glycolysis for energy, and excessive lactate must be transported by MCTs for tumor cell survival [9], while the lactic acid production is harmful to most normal host cells [10,11].

The role of CD147 in infections by pathogens such as human immunodeficiency virus (HIV), hepatitis B (HBV) and C viruses (HCV) and Kaposi’s sarcoma-associated herpesvirus (KSHV) has also been widely studied, revealing CD147-associated mechanisms in viral pathogenesis and tumorigenesis. In particular, CD147 deregulation may lead to a detrimental cycle of disease progression. In addition to its metalloproteinase-inducing ability, CD147 regulates several distinct functions, including spermatogenesis [12,13,14], lymphocyte responsiveness [15] and expression of the MCT system [16]. CD147 released in microvesicles participates in uterine pathological processes, brain edema and malignant plasma cell proliferation [17,18,19,20].

This comprehensive review will provide a detailed overview of the discovery, characterization, molecular structure and diverse biological functions of CD147. In particular, we will present the most recent research findings regarding the bioactivity and regulation of CD147 in human physiological and pathological processes. This review will also provide background on recent studies showing the potential utilization of CD147 as a diagnostic biomarker for different human diseases as well as a target for innovative therapies using CD147-directed monoclonal antibodies and related biologics.

## 2. Discovery and Molecular Characterization of CD147

### 2.1. Discovery and Molecular Structure of CD147

CD147, or basigin, is also known as HAb18G in humans [21]. In contrast, CD147 is referred to as gp42 in mice [5], OX47 in rats [22], and 5A11, HT7 or neurothelin in chickens [23]. CD147 was first named tumor cell-mediated collagen enzyme activation factor (tumor cell collagenase stimulatory factor, TCSF) and then renamed EMMPRIN [4].

**Figure 1:**
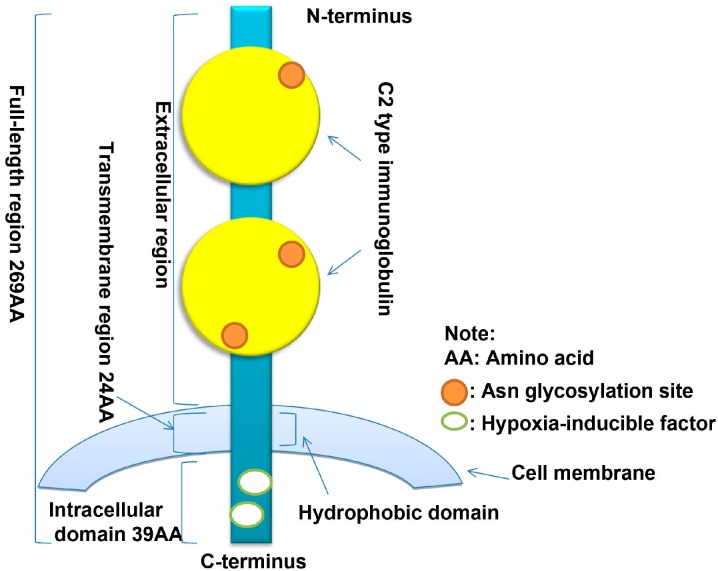
The molecular structure of CD147.

In chromosomal mapping studies, the *CD147* gene has been localized to chromosome 19p13.3 and contains 1797 bp [24,25]. In the 5' region of the *CD147* gene, there is a 30 bp element from −142 to −112 bp that contains binding sites for specificity protein 1 (Sp1), AP1TFII and early growth response-2 (EGR-2), which are important for CD147 transcription [24]. The mouse *CD147*/*basigin* gene consists of approximately 950 bases and is highly conserved. This gene also contains three Sp1 sites and two apetala 2 (AP2) transcription factor consensus binding sites in the 5'-flanking region [26]. The CD147 coding region encodes 269 amino acid residues, including two C2-type immunoglobulin regions in the extracellular *N*-terminal sequence [27], 24 amino acid residues located in the transmembrane region and 39 amino acid residues in the *C*-terminal intracellular region [25]. There are also two hypoxia-inducible factor (HIF) binding sites in the 3'-flanking region of the *CD147* gene [11] (see Figure 1). Additionally, there are 21 highly conserved amino acid residues in the hydrophobic domain structure of the CD147 transmembrane region, which serve as both a signal peptide for CD147 and a cell membrane anchor [22]. The CD147 structure also combines with other proteins for common signal transduction for physiological functional regulation, such as that involving integrin α3-β1 [28] α6-β1 [29] and MCT1 [30]. Two immunoglobulin-like structures in the extracellular region of CD147 activate MMPs [31,32]. Moreover, MMPs secreted via CD147 stimulation also cleave CD147 from the membrane, thereby forming a positive feedback loop [33]. However, the functions, activities and interactions of these MMPs remain largely unknown. Finally, there are three Asn glycosylation sites in the CD147 extracellular region [34]. Treatment of CD147 with glycosidase F generates proteins with molecular masses ranging from 28,000 to 60,000 daltons, which indicates that the *N*-terminus of CD147 is highly glycosylated [26,35]. Dimerization in the CD147 crystal structure plays an important role in allowing CD147 to take part in tumor cell invasion and MMP-2 production [36].

### 2.2. Other Isoforms of CD147

A second isoform of CD147, called CD147 Ig0–Ig1–Ig2, which contains one additional immunoglobulin-like domain in its extracellular portion, has also been characterized [37,38]. Furthermore, the structure and the explicit biological function of the CD147 Ig1–Ig2 domain and CD147 Ig0 domain have been investigated. The specific extracellular forms derived from the primary CD147 isoform are referred to as CD147 Ig1–Ig2 [39]. Specifically, the extracellular forms of CD147 stimulate their own expression, and high levels of both MMPs and pro-inflammatory cytokines are continually secreted due to these extracellular CD147 forms [39,40,41]. CD147 has been detected primarily within the eye as both a cellular and extracellular protein and is implicated in normal retinal development as well as retinoblastoma (RB) [42,43]. Recently, Huang *et al.* [44] found a new isoform of CD147, called EMMPRIN-2, which is the main isoform in head and neck squamous cell carcinoma (HNSCC). EMMPRIN-2 may also promote MMP-2 and urokinase-type plasminogen activator (uPA) to modulate HNSCC invasion and migration. However, the CD147 Ig0 domain alone stimulates interleukin (IL)-6 secreted from HEK293 cells in a dose-dependent manner, in contrast to the other two Ig-like domains. Thus, CD147 Ig0 might be a potent stimulator of IL-6. CD147 Ig0 may have its own special receptor distinct from that of other CD147 Ig-like domains, but the specific receptor has not been identified [38]. Additionally, the CD147 Ig0 dimer is the functional unit required for activity and disrupted by a single point mutation [38]. Moreover, NMR has shown that the CD147 Ig0–Ig1–Ig2, CD147 Ig1-Ig2 and CD147 Ig0 domains do not interact with each other [38], so there might exist some indirect interactions, but the mechanism(s) is unknown. Importantly, naturally soluble forms of CD147 have been discovered in ocular fluids, synovial fluids, HEp-2 human laryngeal epidermoid carcinoma cells and human platelets or plasma [42,45,46,47]. It should be further confirmed whether the roles of these soluble forms resemble the known transmembrane protein functions.

## 3. The Expression and Role of CD147 in Tumor Cells

### 3.1. CD147 Is Over-Expressed in Common Tumors

CD147 is commonly over-expressed in many tumors and is one of the most highly expressed proteins in disseminated cancer cells [48]. Many different human cancers over-express CD147 on tumor cell surfaces, including several common cancers (Table 1) and giant cell tumors [49], synoviocytes [50], cancer stem cells (CSCs) [51], and human multiple myeloma cell lines (HMCLs) [52]. Other investigators have found that CD147 is expressed in the membranous and/or cytoplasmic compartments of ovarian carcinoma [9,53]. Moreover, the CD147 expression level is not related to the age of the cancer patient, tumor type or gross morphology but is related to tumor histopathologic type and clinical stage of disease [52,54]. Interestingly, CD147 expression is often concurrent with other factors involved in the pathological process, such as MCTs. Good colocalization of CD147 and MCT1 has been found in 23 carcinomas by identical immunostaining scores [9]. Furthermore, an immunohistochemical examination of the plasma membranes of MLS-175 soft tissue sarcoma cells revealed that 40% of samples were positive for CD147 and 60% were positive for MCTs (MCT1 and MCT4) [55]. Thus, understanding CD147 biology, which includes identifying the extracellular forms that exist, the MMPs and cytokines that they activate, their cellular targets, and the proteins with which they interact, will have a significant impact on our understanding of cancer biology.

**Table 1:** Dysregulation of CD147 has been associated with nearly every type of cancer.

Cancer	Regulatory Functions of CD147	Investigators (References)
Brain Cancer (Gliomas)	CD147 expression contributes to glioma invasion and metastasis via stimulating MMPs.	Riethdorf *et al.* 2006 [56], Tian *et al.* 2013 [57].
Breast Cancer	CD147 stimulates MMP-2 from fibroblast and regulates breast cell Invasiveness through interacting with P-glycoprotein, CD44, or EGFR.	Taylor *et al.* 2002 [41], Yang *et al.* 2006 [58], Wang *et al.*2008 [59], Grass *et al.* 2013 [60].
Cervical Cancer/Carcinoma	CD147 expression correlated with MCT1 and MCT4 regulates invasion and metastasis and chemosensitivity in human cervical cancer cells.	Ju *et al.* 2008 [61], Pinheiro *et al.* 2009 [62], Zhang *et al.* 2013 [63].
Colon Cancer	CD147-mediated tumor-host interactions regulate colon cancer growth.	Abraham *et al.* 2008 [64].
Endometrial Cancer	CD147 may reduce e-cadherin level and increase vimentin and snail levels in endometrial cancer.	Nakamura *et al.* 2012 [65].
Head and Neck Squamous Cell Carcinoma	CD147 expression mediated by FGFR promotes HNSCC proliferation and metastasis.	Rosenthal *et al.* 2005 [66], Liu *et al.* 2011 [67], Sweeny *et al.* 2012 [68], Knowles *et al.* 2012 [69], Sweeny *et al.* 2013 [70].
Lymphoma	CD147 and LYVE-1 may cooperate to regulate chemoresistance in primary effusion lymphoma.	Qin *et al.* 2011 [71].
Liver Cancer (Hepatocellular Carcinoma)	CD147 overexpression stimulates MMP production, modulates HCC growth and promotes invasion and metastasis; upregulates anoikis resistance of HCCs via interacting with GnT-Iva or Sp1 or Annexin A2.	Mamori *et al.* 2007 [72], Kong *et al.* 2011 [73], Fan *et al.* 2012 [74], Ke *et al.* 2012 [75], Feng *et al.* 2013 [76], Zhang *et al.* 2013 [77], Zhu *et al.* 2014 [78].
Lung Cancer	CD147 regulates the invasion and metastasis of human lung cancer and correlates with HO-1 or Sp1 in NSCLC.	Kong *et al.* 2010 [79], Ke *et al.* 2012 [10], Tsai *et al.* 2012 [80], Xu *et al.* 2013 [81].
Melanoma	CD147 regulates calcium signaling and hypoxia-induced MMP-2 activities via interacting with calcium-modulating cyclophilin ligand for human melanoma progression.	Long *et al.* 2013 [82], Zeng *et al.* 2014 [83].
Oral Squamous Cell Carcinoma	CD147 promotes epithelial-to-mesenchymal transition by activating MMPs for OSCC invasion and progression associated with oxidative stress marker Keap1.	Huang *et al.* 2013 [84], Richard *et al.* 2013 [85], Siu *et al.* 2013 [86].
Ovarian Cancer	CD147 as a partner of MCT1 is overexpressed under the hypoxic microenvironment and mediates cell proliferation and cycling, apoptosis, migration and invasion via activating VEGF and MMP-9 secretion, and vesicles shed from ovarian cancer cells to induce proangiogenic activities of HUVECs.	Millimaggi *et al.* 2007 [87], Fukuoka *et al.* 2012 [9], Yang *et al.* 2013 [11,88], Zhao *et al.* 2013 [89].
Pancreatic Cancer	CD147 as a novel upstream activator of STAT3 interacting with CD44s is highly expressed and plays a critical role in pancreatic cancer development.	Riethdorf *et al.* 2006 [56], Li *et al.* 2013 [90], Sugyo *et al.* 2013 [91].
Retinoblastoma	CD147 plays a role in the up-regulation of MMP-2 in invasive retinoblastoma.	Mӓӓttӓ *et al.* 2006 [42], Adithi *et al.* 2007 [43].
Urothelial Carcinoma of the Bladder	CD147 expression regulates UCB invasion by affecting MMP-2, MMP-9, MMP14 and VEGF secretion. MCT1 and MCT4 may take part in this process.	Wittschieber *et al.* 2011 [92], Xue *et al.* 2011 [93], Bhagirath *et al.* 2012 [94], Choi *et al.* 2014 [95].
Stomach/Gastric Cancer	CD147 expression mediates gastric cancer cell proliferation and invasion via the ERK1/2 signaling pathway and is up-regulated in gastric cancer lesions in correlation with ADAM17.	Shou *et al.* 2012 [96], Chen *et al.* 2013 [97].

ADAM17: a disintegrin and metalloproteinase 17; EGFR: epidermal growth factor receptor; ERK: extracellular signal-regulated kinase; FGFR: fibroblast growth factor receptor; GnT-Iva: acetylglucosaminyltransferase-Iva; HO-1: heme oxygenase-1; HUVEC: human umbilical vein endothelial cells; LYVE-1: lymphatic vessel endothelial hyaluronan receptor-1; NSCLC: non-small cell lung cancer; OSCC: oral squamous cell carcinoma; STAT3: Signal transducer and activator of transcription 3; UCB: urothelial carcinoma of the bladder; VEGF: vascular endothelial growth factor.

### 3.2. The Roles of CD147 in the Invasion, Growth and Metastasis of Different Tumors

CD147 is central in the promotion of tumor invasion, growth/progression and metastasis because it up-regulates MMPs secreted from adjacent fibroblasts [98] through mostly unknown mechanisms. MMPs stimulated by CD147 degrade the extracellular matrix [99] and underlie tumorigenesis, while many of the proinflammatory cytokines and other mediators production stimulated by CD147 have also been directly linked to cancer [52,76]. In fact, the dysregulation of CD147 has been linked to almost every type of cancer (see Table 1). CD147 expression is important to HCC growth and invasion and metastasis via modulating MMP production. Native CD147 purified from HCC cells elevates the production levels of MMP-2 and MMP-9 by stimulating human fibroblasts [100]. In a coculture system, the invasion ability of HCC cells with co-silenced MMP-2 and MMP-9 genes cocultured with fibroblasts is significantly weaker than that of both CD147-silenced HCC cells and MMP-2- and MMP-9-silenced fibroblasts [101]. MMP-2 and MMP-9 secretion are decreased and the migration and invasion of HCC cells are inhibited when using the CD147 antibody, siRNA or other methods such as arsenic trioxide to down-regulate CD147 and MMP-2 [102]. CD147 can inhibit starvation-induced autophagic cell death in SMMC-7721 cells by down-regulating Beclin1 [103]. Chen *et al.* [18] have also found that only insulin-like growth factor-I (IGF-I) markedly up-regulates CD147 expression at the mRNA level and protein levels in SMMC-7721 cells in an IGF-I dose-dependent manner. The ability of proliferation, migration and formation of tube-like structures of HUVECs is significantly enhanced in tumor-conditioned medium (TCM) of CD147-expressing SMMC-7721 cells with IGF-I, but when IGF-I is removed from TCM or the cells are transfected with specific CD147 siRNA, the inductive effect on HUVECs is decreased [18]. In OSCC [84,86], laryngeal carcinoma [104], or NSCLC [105], the role of CD147 is to regulate MMP secretion and control tumor invasion and metastasis, as in HCC. Moreover, CD147 over-expression plays an important role in the development of squamous cell carcinomas [106]. Yang *et al.* [107] successfully constructed a CD147 lentiviral expression vector and stably transfected the A549 cell line (human lung adenocarcinoma cells), achieving CD147 over-expression; they then assessed the *MMP-9* mRNA expression and the proliferation and invasive ability of A549-CD147 cells. The results revealed that the expression level of MMP-9 was increased after CD147 over-expression and that proliferation and invasive ability were strengthened in A549-CD147 cells. When CD147 is silenced in ovarian carcinoma cells, both VEGF and MMP-9 mRNA or protein are down-regulated, while CD147 expression negatively affects basic fibroblast growth factor (bFGF) expression [89,108]. CD147 induces membrane vesicles secreted from embryonal carcinoma NT2/D1 cells and stimulates MMP-2 production in fibroblasts to promote embryonal carcinoma invasion [109]. CD147 may also take part in the up-regulation of MMP-2 in invasive RB [43]. CD147 also confers cancer cell resistance to anoikis through Bim inhibition and is critical for CSC chemoresistance [51,58]. In thyroid medullary carcinoma TT cells, CD147 siRNA inhibits cell proliferation but does not significantly affect the apoptosis, migration or invasion of the TT cells, because the mRNA and protein of MMP-2 are decreased and the cell cycle is changed [110]. However, there exists a different view that in several cancer cell lines, MMP production may be not regulated by CD147 (not published).

Some investigators have focused on the role of CD147 in the pathogenesis of KSHV, a common etiology by which cancers can achieve immune suppression [111,112,113,114]. The ectopic expression of KSHV-encoded latency-associated nuclear antigen (LANA) significantly increases the expression of high-MW glycoform CD147 following *de novo* infection of endothelial cells (EC), and CD147 over-expression promotes EC invasion by up-regulating VEGF [113]. This observation was the first evidence that KSHV up-regulates CD147. Meanwhile, when CD147 is knocked down, KSHV-initiated VEGF and IL-6 secreted from EC are significantly reduced [114]. Based on these data, CD147 is a co-factor in the infection and pathogenesis of oncogenic viruses. However, no data support the direct regulation of CD147 by oncogenic viruses. In future studies, we should determine if targeting CD147 directly would selectively disrupt the pathogenesis of KSHV-associated cancer.

### 3.3. CD147 Always Associates with Other Proteins in Tumors

In cancer pathological processes, CD147 always interacts with other proteins to affect tumor cell invasion and metastasis. The known CD147-interacting partners include MCT1, integrin-β1, cyclophilin, and Ubiquitin C, among others. Western blot analysis has further revealed that the down-regulation of MCT1 also down-regulates the CD147 protein, despite having no effect on CD147 mRNA levels. The association between MCT1 and/or MCT4 and CD147 is involved in lactate export and proliferation in HMCLs [52]. A novel interaction between the calcium-modulating cyclophilin ligand and CD147 regulates calcium signaling and MMP activity in human melanoma cells [82]. Human tumor cells induce angiogenesis through positive feedback between CD147 and insulin-like growth factor-I [18]. Annexin A2 also co-localizes and co-immunoprecipitates with CD147, which may enhance the migration and invasion potential of HCC cells *in vitro* by regulating the trafficking of CD147-harboring membrane microvesicles and MMP-2 production [77]. CD147 and anterior gradient homolog 2 (AGR2) expression promotes cellular proliferation and metastasis in HNSCC [68]. In addition, the ubiquitination interaction between CD147 and P-glycoprotein in breast cancer cells represents a regulatory mechanism of metastasis [59]. Additionally, CD44 and CD147 have been associated with the metastasis and progression of breast or prostate cancer [60,115]. One study has demonstrated that the expression of COX-2 and CD147 is significantly increased in hypopharyngeal squamous cell carcinoma (HSCC) tissues compared to the adjacent epithelium, and these authors have also reported a strong correlation between COX-2, CD44v6 and CD147 expression and invasion and lymph node metastasis in HSCC that is associated with T classification, lymph node metastasis and clinical stage [116]. Recent data have further shown that the up-regulation of CD147 in non-transformed, non-invasive breast epithelial cells is sufficient to induce an invasive phenotype characterized by membrane type-1-MMP-dependent invadopodium activity [117].

## 4. The Expression and Role of CD147 in Tissues and Diseases Other than Cancer

### 4.1. CD147 Expression in Healthy Tissues and Other Diseases

CD147 plays an important role in a number of organ systems, including the Ok blood group [118], cardiovascular system [119,120], nervous system [121], and T cells of the immune system [122]. CD147 is expressed in epithelial cells [99], fibroblasts [123], psoriatic peripheral blood mononuclear cells [124], cytotrophoblasts [125], the normal basal epithelial layer reportedly harboring stem cells [126], ectopic endometrial tissues [127], systemic lupus erythematosus [128], synovial joint disease tissue of rheumatoid arthritis (RA) patients [115,129], plasma of lupus nephritis patients [130], peripheral monocytes and T lymphocytes of patients with ankylosing spondylitis [131].

### 4.2. Roles of CD147 in Tissue Systems or Other Diseases to Promote MMP Production

In previous research on experimental autoimmune encephalomyelitis in mice [132], CD147 was found to be involved in the matrix metalloproteinase-mediated cell migration across the parenchymal basement membrane into the central nervous system (CNS) parenchyma. Another study has also shown that CD147 has a crucial role in leucocyte adhesion to endothelial cells, the first step in immune cell migration into the CNS. In multiple sclerosis brain sections with inflammation, CD147 blockage reduces α 4 integrin expression on T cells, due to its inhibition of nuclear factor κB (NFκB) translocation to the nucleus for gene transcription [122]. CD147 has additional roles in leucocyte adhesion by affecting α 4 integrin expression through the NFκB signal pathway. CD147 also plays an essential role in germ cell migration and survival/apoptosis during spermatogenesis [14]. In acute coronary syndrome patients with unstable coronary artery plaques, CD147 expression is increased on macrophages (Mφ) and smooth muscle cells, but CD147 genetic polymorphisms are not an important factor in atherosclerotic cerebral infarction in the Han Chinese population [133,134]. CD147 is also associated with the formation of brain edema induced by subarachnoid hemorrhage (SAH) in the monofilament puncture model of male Sprague–Dawley rats [19], but the direct role of CD147 and its mechanism of action in SAH need further study.

CD147 regulates MMPs in a variety of physiological and pathological conditions. In biopsy tissue, the rheumatoid synovium and adjacent fibroblast cells obtained from RA patients produce numerous MMP family members [35,135], and these proteins differentially regulate cell–cell recognition between different immune cell types, as well as cell differentiation in the bone and cartilage. The expression level of CD147 on CD14^+^ monocytes in RA patients of damp-heat bi-syndrome (DHBS) is much lower than that in cold-damp bi-syndrome and normal controls; otherwise, the level of serum MMP-3 and the MFI of CD147 are higher in DHBS [129]. In liver fibrosis and cirrhosis, transforming growth factor-β1 (TGF-β1) stimulates CD147 overexpression, which induces collagen I and MMP-2 secretion to promote hepatic stellate cell activation, while CD147 expression decreased during spontaneous recovery from liver fibrosis [123]. CD147 also promotes lung tissue fibrosis. In a murine bleomycin-induced lung interstitial fibrosis model, CD147 induces Th17 cell differentiation and the secretion of some cytokines from M1 Mφ [136]. In terms of uterine pathology, CD147 expression is increased by G protein-coupled receptor 30 (GPR30), estradiol or cholera toxin stimulation in a human uterine epithelial cell line, subsequently promoting MMP production [17].

### 4.3. CD147 Interacts with Other Proteins to Regulate Physiological and Pathological Processes

In normal physiological processes or diseases other than cancers, CD147 is associated with MCTs and serves as a receptor for other proteins that interact with CD147 to carry out their functions. MCTs, as essential nutrients, are important to retinal pigmented epithelium (RPE) development. In mouse retina, a complex formed by CD147 and MCTs appears in the plasma membrane. Furthermore, when CD147 is knocked down, the mouse loses sight because nutrient transfer is stopped in the RPE as a result of MCTs not integrating with the membrane [137]. Using isolated rat hearts to detect the expression levels of MCTs and their ancillary protein CD147, Zhu *et al.* found MCT4 expression increased in global ischemia, MCT1 expression enhanced in the early stages of reperfusion, and CD147 expression increased during ischemia-reperfusion injury [30]. Moreover, Kanyenda *et al.* [121] have reported that increased CD147 expression in rat cortical neuronal cultures protects against Aβ42 (amyloid-β) toxicity, but only when recombinant Cyp-A protein is added to the neuronal cultures. CD147 exists as a receptor on human trophoblast cells and regulates the implantation, invasion and differentiation of trophoblasts, as demonstrated using siRNA or anti-CD147 functional blocking antibodies to suppress *CD147* gene expression and protein levels. Although the mechanism by which reducing CD147 expression in placenta leads to pre-eclampsia is unclear, it has been speculated that inhibition of the enzymatic activities of MMP-2, MMP-9 and uPA might cause this effect [125]. In mouse fetal tissue, CD54^+^ MD may recruit and locally activate other Ma to express CD147 and CD169 until the day before C3H/HeN mice are born [138]. Finally, CD147 has been identified as the erythrocyte receptor of PfRh5 and is essential for the invasion of multiple strains of *Plasmodium* parasites, including the species that cause human malaria [139,140,141]. Notably, PfRH5 binding to CD147 is a species-specific host tropism of *P. falciparum*, which prefers human hosts [142]. CD147 is a critical host receptor for PilE and PilV (meningococcal pilus components) in the pathological process of *Neisseria meningitides*, which quickly causes fatal septic shock [143]. Recently, CD147 and Semaphorin-7A (CD108) have been identified as novel *P. falciparum* erythrocyte invasion receptors for RH5 and the merozoite-specific thrombospondin-related anonymous protein, respectively, by using systematic extracellular protein interaction screens [144]. It is believed that malaria vaccines associated with CD147 and/or Rh5 effectively generate antibodies to the parasite.

## 5. Recent Studies on the Regulatory Mechanism of CD147

**Figure 2:**
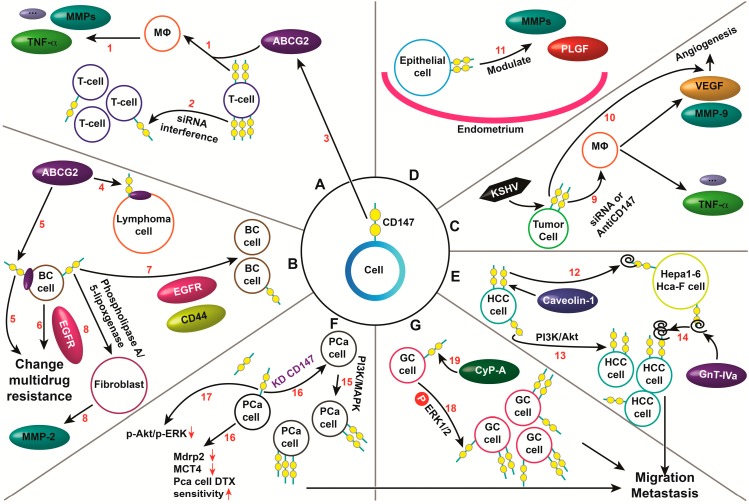
CD147 plays an important role in a variety of cells and tissues regardless of health or disease. (**A**) T cell-mediated immune response; (1) The regulatory role of CD147; (2) The “negative” regulatory role of CD147 as shown by CD147 knockdown (KD), leading to increased T cell proliferation; (3) CD147 regulates the ABCG2-driven transport of methotrexate; (**B**) Regulating the expression, dimerization and drug transporter function of ABCG2; (4) CD147–ABCG2 complex on the lymphoma cell membrane; (5) CD147 and ABCG2 mediate multidrug resistance in breast cancer (BC); (6) EGFR promotes multidrug resistance; (7) CD147, CD44 and EGFR signaling pathways cooperate to regulate invasiveness; (8) The phospholipase A_2_- and 5-lipoxygenase-catalyzed pathway and CD147 stimulate fibroblast MMP-2 release; (**C**) Tumor cell-Mφ interactions promote angiogenesis; (9) CD147 enhances the monocytic secretion of MMP-9 and VEGF, while inhibition of its expression decreases the induction of these two pro-angiogenic proteins; (10) KSHV activation of CD147 induces P13K/Akt- and MAPK-dependent secretion of VEGF; (**D**) The effects of CD147 on trophoblastic function; (11) Under progesterone-rich conditions, CD147 regulates MMPs and PLGF during gestation; (**E**) Regulating the proliferation, anoikis, migration and metastasis of HCCs; (12) Caveolin-1 promotes HCC invasion by up-regulating the glycosylation of CD147; (13) CD147 mediates resistance to anoikis in HCC cells by activating the PI3K/Akt pathway; (14) The key role of GnT–IVa in the migration and metastasis of HCCs involves alteration of the antennary oligosaccharide structures on glycosylated CD147; (**F**) Regulating the metastasis and chemoresistance of PCa cells; (15) CD147 expression is mediated by the PI3K and MAPK pathways; (16) CD147 KD decreases MCT4 and multi-drug resistance protein-2(Mdrp2) expression and reduces the invasive potential and proliferation of PCa while enhancing docetaxel sensitivity; (17) CD147 KD down-regulates p-Akt and p-Erk, the main signal modulators associated with cell growth and survival; (**G**) Regulating the migration and metastasis of GCs; (18) The ERK1/2 signaling pathway is involved in CD147-mediated proliferation and invasion of gastric cancer cells; (19) Extracellular Cyp-A stimulates ERK1/2 phosphorylation.

As mentioned above, CD147 plays an important role in a variety of cells and tissues in both health and disease. Indeed, CD147 regulates the immune response, cancer chemoresistance, cellular proliferation and anoikis, and tumor cell migration, metastasis and differentiation (Figure 2). Importantly, recent studies have revealed novel signaling pathways to explain the regulatory mechanisms of CD147 in different cancers. Regarding immune response regulation, Biegler *et al.* [32] have declared a possible “negative” regulatory role for CD147 in T cell-mediated immune responses because knocking down the *CD147* gene increases, rather than decreases, T cell proliferation (Figure 2A).

### 5.1. CD147 Takes Part in the Regulatory Mechanism of Chemoresistance

Regarding the regulation of cancer chemoresistance, there are some reports that CD147 regulates certain proteins, e.g., ATP-binding cassette transporter G2 (ABCG2) and EGFR, to mediate chemoresistance. CD147 regulates ABCG2-mediated transport of the cytotoxic agent methotrexate in immune cells [124], although this mechanism remains poorly characterized. CD147 can form a complex with ABCG2 on the cell membrane in primary effusion lymphoma and breast cancer cells, as determined by co-immunoprecipitation [71,145]. Specifically, CD147 can increase ABCG2 protein expression, but not mRNA expression, and affect the cellular localization and dimerization of ABCG2, thereby regulating its drug transporter function in MCF-7 breast cancer cells (Figure 2B) [145]. However, the relationships between CD147 and ABCG2 are not clear. Whether they interact via the PI3K/Akt signaling pathway or whether CD147 indirectly up-regulates ABCG2 needs further study. Additionally, the CD147, CD44, EGFR and MCT signaling pathways cooperate to regulate breast epithelial cell invasiveness [60,146]. CD147 stimulates hyaluronan production to regulate multidrug resistance in cancer cells, with hyaluronan often binding to CD44 for the coregulation of signaling pathways [147]. Furthermore, CD147 and LYVE-1 coregulate the chemoresistance of KSHV-infected lymphoma cells [71]. Using a neutralizing antibody to uPAR could down-regulate the HNSCC resistance to cisplatin induced by CD147 over-expression, resulting in increased HNSCC sensitivity to cisplatin, which may indicate that CD147 and uPAR collaborate in the chemoresistance process [148]. The inhibition of CD147, P-gp, and p-Akt by STAT3 decoy oligodeoxynucleotide (ODN) technology inhibits ovarian cancer cell invasiveness and enhances the paclitaxel sensitivity of SKOV3 and OVCAR3 ovarian cancer cells [149].

### 5.2. CD147 Regulates MMP and VEGF Production or Signals for Tumor Cell Invasion and Metastasis

CD147 secreted from breast cancer cells induces MMP-2 release from fibroblasts mediated by the activation of a phospholipase A2- and 5-lipoxygenase-catalyzed pathway in fibroblasts [41]. Furthermore, CD147 over-expression enhances phosphorylation of PI3K, Akt and ERK, while the inhibition of PI3K, Akt or mitogen-activated protein kinase (MAPK) activation significantly suppresses CD147-induced VEGF secretion and invasion of HUVECs [114]. This observation indicates that KSHV-induced VEGF secretion and endothelial cell invasion are mediated through KSHV’s up-regulation of CD147 and activation of the PI3K/Akt and MAPK pathways (Figure 2C). In the KS microenvironment, latent KSHV infection of human primary oral fibroblasts induces a tumor-associated fibroblast-like phenotype for these cells via KSHV regulation of CD147 transcription [112]. In tumor cells co-cultured with Mφ, CD147 expression is increased and required for maximal induction of both MMP-9 and VEGF secreted from Mφ. In addition, CD147 may effect pro-angiogenesis in endothelial cells by the triage of tumor cell-Mφ-endothelial cell interaction [150]. CD147 regulates several VEGF isoforms and placental growth factor (PLGF) [151], and it has unique effects on trophoblastic function (Figure 2D). CD147 from epithelial cells modulates bovine endometrial cell functions during gestation by regulating MMPs in the endometrium, and some ovarian steroids, such as progesterone, can increase CD147 expression [152,153]. Finally, CD147 has a role in CSC growth and survival. CD147 gene silencing by siRNA can restore the sensitivity of CSC-like cells derived from MDA-MB453 breast cancer cells to 5-fluorouracil and increase p-glycogen synthase kinase 3β expression while reducing the expression of thymidylate synthase, p-Akt, and β-catenin [51].

The roles of CD147 in the migration, metastasis and resistance to anoikis of tumor cells involve the up-regulation of some unique proteins and special signal transduction pathways. Caveolin-1 (a major structural protein of caveolae) can promote cell invasion in mouse HCC Hepa1-6 and Hca-F cells by up-regulating CD147 glycosylation (Figure 2E) [34]. CD147 mediates HCC cells’ resistance to anoikis, which is partially dependent on the activation of the PI3K/Akt pathway (Figure 2E) [75]. The *N*-GnT–IVa may play a key role in the migration and metastasis of mouse HCCs by altering the glycosylation of CD147, as this enzyme can significantly change the antennary oligosaccharide structures on CD147 [74]. Some investigators have therefore speculated that this finding represents a new mechanism underlying the metastasis of HCC through direct effects on CD147.

Other *in vitro* and *in vivo* studies have revealed novel signaling pathways in different cancers. For example, prostate cancer (PCa) metastasis and chemoresistance may be modulated by the increased expression of either CD44 or CD147 as a result of activation of the PI3K and MAPK pathways (Figure 2F) [115]. Knocking down either CD44 or CD147 along with multi-drug resistance protein-2 (MRP2) and transporter protein (MCT4) reduces both p-Akt and p-Erk in PCa cells, while docetaxel sensitivity is increased, which affects PCa metastasis and chemoresistance [115]. The ERK1/2 signaling pathway (Figure 2G) is involved in CD147-mediated gastric proliferation and invasion of the gastric cancer cell line SGC7901 [97]. Extracellular Cyp-A may be involved in stimulating ERK1/2 phosphorylation and NF-κB [154].

### 5.3. Some New Findings on CD147 Regulatory Mechanisms in Other Diseases via Signal Pathways

Importantly, CD147 is involved in the activation of NF-κB signaling for inflammation in monocytes and RA [155,156]. New findings have suggested that in the synovial joints of RA patients, there are interactions between extracellular Cyp-A from fibroblast-like synoviocytes and CD147 expressed in Mφ, which may promote arthritis development [135]. Cyclosporine also inhibits the expression of CD147 to affect the expression of MCT1, because CD147 regulates MCTs by binding to cyclophilins and FKBPs [157,158,159]. Cyclosporine A-induced downregulation of CD147 is accompanied by a decrease in the activated (phosphorylated) forms of IKKγ and p65 in response to salt depletion in nephrotoxicity-exposed rats [158]. Finally, CD147 is a potent inducer of IL-18 mRNA and protein expression in adult mouse cardiomyocytes (ACM), primarily by inducing NF-κB and AP1 binding to the IL-18 promoter via Rac1-mediated PI3K/Akt/IKK-dependent IκB-α degradation and MKK7/JNK-dependent AP1 activation [160]. These results suggest that CD147 and IL-18 together may form a mutually reinforcing response mechanism to myocardial injury, leading to adverse myocardial remodeling. CD147 also activates cyclic AMP response element-binding protein and activating transcription factor-2 and regulates the expression of both MMPs and tissue inhibitors of the matrix metalloproteinases (TIMPs) from ACM in a time-dependent manner, but the mechanism is not known [160].

## 6. Additional Regulatory Mechanisms Controlling CD147 Expression and Function

Additional factors affect CD147 expression, including hypoxia-induced cellular microenvironments, transcription factors, estradiol, hormones and GPR30. Several cytokines and proteins correlate with the up-regulation of CD147, including IGF-I [18], TGF-β1 [123,161,162], EGF [163], cPLA2ε [164], galectin-3 [165] and leukotriene B4 (LTB4) [133]. CD147 expression is up-regulated in hypoxic regions of epithelial solid tumor tissues. Other studies on the metabolic functions of hypoxia-induced CD147 have found that CD147 promotes glycolysis in tumor cells of a nude mouse xenograft tumor model, partially through functional cooperation with MCT1 and MCT4 [10,11]. Because CD147 is the partner of the MCTs, CD147 plays a determinant role in mediating glycolysis for tumor growth, as shown via CD147 knockdown in human colon adenocarcinoma LS174T cells or MCTs in Ras-transformed fibroblast CCL39 cells [166]. When CD147 is knocked down, lactate transport is arrested because intracellular lactate may not be exported from the cell by MCTs. Moreover, blocking CD147 and integrin-β1 on RPE cell surfaces inhibits the binding of galectin-3, which inhibits the attachment and spreading of RPE cells, suggesting that galectin-3 acts as a positive regulator of CD147/integrin-β1 clustering [167]. In recent years, the regulatory roles of transcription factors in the expression of CD147 have received specific attention, including the functions of Sp1, EGR-2 and HIF-1. Investigators have identified a critical Sp1 binding site located at −87 to −81 and have confirmed its essential role in up-regulating CD147 promoter activity [79]. This result provided the first evidence that HIF-1 directly binds to a specific hypoxia-responsive element (HRE) located at −133 to −130 in the CD147 promoter region [10]. Moreover, in hypoxia-induced cellular microenvironments, the transcription factors HIF-1 and Sp1 have a combined effect on activation of the CD147 promoter, and the activation of these transcription factors leads to the over-expression of CD147 on the surface of tumor cells [10,11]. In particular, demethylation with 5-aza-2'-deoxycytidine increases CD147 expression in HCCs by increasing Sp1 binding affinity; furthermore, the recurrence rate and death rate of HCC patients with an unmethylated CD147 promoter are higher than those of patients with a methylated CD147 promoter [73]. Expression of the HCV core protein contributes to the metastasis of hepatocyte cells by promoting Sp1 binding to activate the transcription of CD147 [168]. The transcription factor Sp1 also regulates CD147 expression in human lung cancer [79]. Moreover, KSHV induces these effects through Sp1- and EGR-2-dependent CD147 transcriptional activation. IL-18 induces CD147 expression in cardiomyocytes via MyD88/IRAK4/TRAF6/JNK-dependent Sp1 activation [169]. Finally, an autoregulatory loop formed by miR-22, Sp1 and c-Myc binding to the CD147 promoter regulates CD147 expression in breast cancer [170]. miR-146a increases CD147 expression in MCF-7 cells, but the same effect does not occur in human renal carcinoma A498 cells [150].

Interestingly, uterine remodeling is highly dependent on CD147. Estradiol and GPR30 can increase CD147 expression in uterine cells [17,18]. Oxidized low-density lipoprotein enhances CD147 expression in human platelets and in coronary smooth muscle cells, whereas high-density lipoprotein or anti-LOX-1 monoclonal antibody decreases CD147 expression [47]. In particular, minocycline intervention significantly reduces the activity of CD147 and MMPs in atherosclerotic plaque tissue, and histology studies have demonstrated enhanced plaque stabilization [171]. Elevated expression of ADAM17 is positively correlated with CD147 expression due to its activation of EGFR in gastric cancer, especially in lesions, compared to adjacent non-cancerous tissues [96]. Redzic and colleagues [161] have demonstrated that extracellular vesicles secreted from MCF-7 and U937 cancer cells stimulate full-length CD147 production. Meanwhile, CD147 may be a general marker of extracellular vesicle secretion. CD147 also interacts with caveolin-1 on the A431 carcinoma cell surface, an association which depends on the second immunoglobulin domain in the extracellular portion of CD147, but seems to negatively regulate the clustering and activity of CD147 to reduce MMP-1 production dependent on CD147 [172]. Real-time PCR studies confirmed that TIMP-1 is increased approximately twofold, while the expression levels of cyclin-dependent kinase 5 and CD147 are decreased approximately twofold, at 24 h after the induction of pinin, a nuclear and desmosome-associated protein [173]. When exogenous HO-1 is over-expressed in NSCLC A549 and H441 cells, EGFR, CD147 and MMP-9 expression are enhanced, whereas they are decreased after HO-1 silencing [80]. However, the exact role of HO-1 in the up-regulation or down-regulation of EGFR and CD147 requires further investigation. CD147 in SMMC7721 cells is markedly down-regulated by baicalein, accompanied by the occurrence of apoptosis and autophagy, but the mechanism or signal pathway that determines this effect is not known [174].

## 7. Clinical Applications of CD147

### 7.1. Potential Diagnostic Markers of Disease

CD147 expression is associated with tumor progression and prognosis. The potential application of CD147 is not only as a marker of activated regulatory T cells [175] but also as a potential diagnostic marker of early stage diseases. The pivotal role of CD147, which reaches beyond that of a mere marker of inflammation, is demonstrated in the complex processes of atherogenesis, atheroprogression and acute atherosclerothrombosis [175]. The extra-endothelial expression of CD147 is a marker of the activity of lesions in multiple sclerosis, as this expression is present in leukocyte-containing perivascular cuffs but not in inactive lesions [122].

Indeed, CD147 has been suggested as a prognostic marker in endometrial cancer [65], gastric cancer [96], glioblastoma [98], HCC [78], lupus nephritis [130], UCB [94] and other diseases. Patients with high CD147 expression have a poor prognosis [104], while multivariate analysis revealed that the expression of CD147 is an independent prognostic factor for patients with NSCLC [81,176]. The five-year survival rate in NSCLC cases with low CD147 expression is higher than that in cases with high expression [176], and CD147 may therefore represent a useful biomarker for prognostic evaluation. Some investigators detected 360 UCB cases by immunohistochemistry to determine the prognostic significance of MCT1, MCT4, and CD147 expression. The results showed that only high MCT1 and CD147 expression were associated with high World Health Organization grade, advanced tumor node metastasis stage and nonpapillary growth type, while high MCT4 expression was not significantly associated with any variable [95]. Thus, CD147, MCT1 and MCT4 help to predict overall survival and recurrence-free survival in univariate or multivariate analyses.

CD147, as a molecular target of ultrasonographic contrast agents, may improve the detection sensitivity and specificity of microbubbles in HNSCC [69]. CD147 may also represent a potential predictor of the degree of malignancy in OSCC [86] and tumor metastasis in HSCC [116]. Boye *et al.* [177] suggest that CD147 could be used in the selection of stage III patients for adjuvant therapy for colorectal cancer, and Yang *et al.* [98] showed that CD147 expression is associated with poor overall survival in patients with glioblastoma and suggest that CD147 may be used to detect treatment response in glioblastoma patients. Together, recent findings suggest that low CD147 expression may be a predictor of favorable prognosis and a prognostic indicator of malignant tumors [76]. Grupp *et al.* [178] concluded that CD147 is not a relevant prognostic biomarker but that it may provide further evidence for marked biological differences between “fusion-type” and “non-fusion-type” PCa.

### 7.2. CD147 as a Potential Therapeutic Target

CD147 may be a promising therapeutic target in cancer and other diseases. For example, CD147 may represent a potential therapeutic target for human cervical cancer and an effective chemotherapy-sensitizing agent [63]. A preclinical study has demonstrated the feasibility of using anti-CD147 microbubble contrast agents for ultrasonographic imaging and for contrast-enhanced imaging of HNSCC tumors *in vivo* [69]. CD147 may also serve as a promising therapeutic target for highly aggressive pancreatic cancer and a surrogate marker in STAT3-targeted molecular therapies [90]. Similarly, PCa patients can benefit from anti-CD147 therapy. Investigators have evaluated the potential of an ^89^Zr-labeled fully human anti-CD147 monoclonal antibody (059-053) as a new positron emission tomography probe for pancreatic cancer in a mouse model [91]. Additionally, the selective targeting of CD44/CD147 alone or in combination with docetaxel may limit PCa metastasis and increase chemosensitivity [115]. Moreover, targeting CD147 may reduce VEGF secretion and EC migration in the KS microenvironment, so it is a novel and potential treatment for KSHV-associated tumors [112,113,114]. In addition, CD147 has been considered in the clinical treatment of psoriatic patients resistant to methotrexate [124], and inhibition of CD147 expression may be a potential treatment of ongoing edema after SAH [19]. Anti-CD147 may prevent the excessive entry of leukocytes into the CNS in conditions for multiple sclerosis therapy [122]. CD147 is a target for antibody therapy for liver fibrosis, owing to its ability to promote the activation of hepatic stellate cells [123]. Finally, CD147 is a potential therapeutic target for adverse myocardial remodeling [160].

### 7.3. Anti-CD147 Antibody Therapeutics

CD147 has been recognized as an effective therapeutic target for HCC [72] and HNSCC [70], and clinical progress has been made in HCC treatment using CD147-directed monoclonal antibodies. Investigators have focused on the role of CD147 in HCC invasion and metastasis, as well as in angiogenesis and multidrug resistance. Compared to saline and ^131^I treatment groups, using a ^131^I-labeled anti-CD147 monoclonal antibody to treat HCC decreased HCC metastasis in a rabbit model. Niu *et al.* [179] suggest that CD147-Ab would serve as a promising drug for the treatment of HCC by inhibiting metastasis and growth, decreasing the expression of MMP-2 and CD31 and inducing tumor necrosis. A novel extracellular CD147 drug conjugate significantly inhibits HNSCC [70]; in particular, EDC22 linked with a small-molecule inhibitor of the Na/K-ATPase was evaluated as an extracellular drug conjugate for the monoclonal antibody targeting of CD147. Sweeny *et al.* [70] concluded that EDC22 is a potent inhibitor of HNSCC cell proliferation *in vitro* and *in vivo*, and these results provide further data on the clinical potential of EDC22 in the treatment of HNSCC. Agrawal *et al.* [180] constructed a novel CD147 antibody to decrease T cell proliferation and neurotoxicity, two factors associated with multiple sclerosis.

CD147 expression is enhanced in Mφ and smooth muscle cells in unstable coronary artery plaques from acute coronary syndrome patients. In addition, LTB4 can stimulate CD147 expression on THP-1-derived Mφ, suggesting that LTB4 and CD147 may both be involved in the formation and progression of unstable plaques [133]. Therefore, future studies should explore whether LTB4 and CD147 antagonists are effective for treating patients with ACS. CD147 has also been recognized as a receptor for malaria infection, which perhaps could contribute to the prevention and treatment of malaria, including anti-CD147 therapies for drug-resistant malaria [141].

## 8. Conclusions

CD147 is a highly glycosylated cell surface type I transmembrane protein [1,3] involved in a range of process, including angiogenesis [114,151], inflammatory diseases and cancer progression. Multiple human pathogens utilize CD147 for efficient infection. CD147 also acts as a hypoxia-responsive molecule. In addition, remarkable heterogeneity in CD147 expression between different tumor types has been observed. For example, the positivity rate of CD147 was 67.76% in epithelium-derived carcinomas, including lung cancer, hepatocellular carcinoma and breast cancer, whereas this rate was only 5.18% in normal epithelial tissues [181]. CD147 promotes tumor invasion and metastasis by stimulating MMP synthesis in neighboring fibroblasts [66] and induces chemoresistance in tumor cells by stimulating them to produce hyaluronan [147]. Indeed, the inhibition of *CD147* gene expression via RNA interference reduces tumor cell invasion and tumorigenicity and increases chemosensitivity to paclitaxel [182]. In several cancers, CD147 expression is so elevated that it is now used as a prognostic biomarker to diagnose early-stage disease and an effective therapeutic target for some cancers. Indeed, exciting clinical progress has been made in HCC treatment using CD147-directed monoclonal antibodies. Although CD147 has been extensively investigated, some questions and uncertainties remain. Whether CD147-Abs cause side effects in the human body requires investigation. Therefore, CD147’s suitability as a potential therapeutic target should be assessed. It is thought that a greater understanding of the physiological and pathological mechanisms of CD147 and other molecular factors involved in disease prognosis will lead to new insights into accurate prognostic prediction, which is critical to the selection of appropriate therapeutic approaches. Moreover, the prognostic role of CD147 could be used to further analyze the overall survival of patients with carcinomas such as glioma and HCC [57,73]. Another potential utility of targeting CD147 is to reduce VEGF secretion and EC migration in the KS microenvironment [114].

Meanwhile, although transmembrane CD147 has been proposed to act as a receptor for several extracellular proteins, such as the cyclophilin class of enzymes [3], CD147 also exhibits ligand activity. This ligand activity leads to the CD147-mediated stimulation of multiple protein families and is thought to underlie the progression of many diseases, such as RA [50], and most cancers, including retinoblastoma [99]. Agents targeting either CD147 or cyclophilin activity show significant anti-inflammatory effects in experimental models, suggesting that CD147-cyclophilin interactions represent dual targets for new anti-inflammatory therapeutics [183], especially for the natural soluble forms present in ocular and synovial fluids. Similar approaches are currently under investigation to develop reagents to inhibit selective functions of CD147, such as the targeting of specific domains of the molecule. Progress in these endeavors will likely provide new treatment opportunities for several inflammatory diseases, including RA, psoriasis and asthma, and may also contribute to the treatment of such diseases as atherosclerosis and cancer. Therefore, novel methods or ideas should be proposed to study CD147, such as the use of double-specific antibodies, which may represent a new direction for treatment.

## References

[1] Simon-Chazottes D., Matsubara S., Miyauchi T., Muramatsu T., Guenet J.L. (1992). Chromosomal localization of two cell surface-associated molecules of potential importance in development: Midkine (Mdk) and basigin (Bsg). Mamm. Genome.

[2] Kasinrerk W., Fiebiger E., Stefanova I., Baumruker T., Knapp W., Stockinger H. (1992). Human leukocyte activation antigen M6, a member of the Ig superfamily, is the species homologue of rat OX-47, mouse basigin, and chicken HT7 molecule. J. Immunol..

[3] Saxena D.K., Oh-Oka T., Kadomatsu K., Muramatsu T., Toshimori K. (2002). Behaviour of a sperm surface transmembrane glycoprotein basigin during epididymal maturation and its role in fertilization in mice. Reproduction.

[4] Biswas C., Zhang Y., DeCastro R., Guo H., Nakamura T., Kataoka H., Nabeshima K. (1995). The human tumor cell-derived collagenase stimulatory factor (renamed EMMPRIN) is a member of the immunoglobulin superfamily. Cancer Res..

[5] Miyauchi T., Masuzawa Y., Muramatsu T. (1991). The basigin group of the immunoglobulin superfamily: Complete conservation of a segment in and around transmembrane domains of human and mouse basigin and chicken HT7 antigen. J. Biochem..

[6] Tyler R.E., Pearce M.M., Shaler T.A., Olzmann J.A., Greenblatt E.J., Kopito R.R. (2012). Unassembled CD147 is an endogenous endoplasmic reticulum-associated degradation substrate. Mol. Biol. Cell.

[7] Warburg O. (1956). On the origin of cancer cells. Science.

[8] Hanahan D.W.R. (2011). Hallmarks of cancer: The next generation. Cell.

[9] Fukuoka M., Hamasaki M., Koga K., Hayashi H., Aoki M., Kawarabayashi T., Miyamoto S., Nabeshima K. (2012). Expression patterns of emmprin and monocarboxylate transporter-1 in ovarian epithelial tumors. Virchows Arch..

[10] Ke X., Fei F., Chen Y., Xu L., Zhang Z., Huang Q., Zhang H., Yang H., Chen Z., Xing J. (2012). Hypoxia upregulates CD147 through a combined effect of HIF-1α and Sp1 to promote glycolysis and tumor progression in epithelial solid tumors. Carcinogenesis.

[11] Yang H., Zou W., Chen B. (2013). Overexpression of CD147 in ovarian cancer is initiated by the hypoxic microenvironment. Cell Biol. Int..

[12] Chen H., Fok K.L., Yu S., Jiang J., Chen Z., Gui Y., Cai Z., Chan H.C. (2011). CD147 is required for matrix metalloproteinases-2 production and germ cell migration during spermatogenesis. Mol. Hum. Reprod..

[13] Fok K.L., Chen H., Ruan Y.C., Chan H.C. (2014). Novel regulators of spermatogenesis. Semin. Cell Dev. Biol..

[14] Chen H., Lam Fok K., Jiang X., Chan H.C. (2012). New insights into germ cell migration and survival/apoptosis in spermatogenesis: Lessons from CD147. Spermatogenesis.

[15] Chiampanichayakul S., Peng-in P., Khunkaewla P., Stockinger H., Kasinrerk W. (2006). CD147 contains different bioactive epitopes involving the regulation of cell adhesion and lymphocyte activation. Immunobiology.

[16] Kirk P., Wilson M.C., Heddle C., Brown M.H., Barclay A.N., Halestrap A.P. (2000). CD147 is tightly associated with lactate transporters MCT1 and MCT4 and facilitates their cell surface expression. EMBO J..

[17] Burnett L.A., Light M.M., Mehrotra P., Nowak R.A. (2012). Stimulation of GPR30 increases release of EMMPRIN-containing microvesicles in human uterine epithelial cells. J. Clin. Endocrinol. Metab..

[18] Chen Y., Gou X., Ke X., Cui H., Chen Z. (2012). Human tumor cells induce angiogenesis through positive feedback between CD147 and insulin-like growth factor-I. PLoS One.

[19] Tu Y., Fu J., Wang J., Fu G., Wang L., Zhang Y. (2012). Extracellular matrix metalloproteinase inducer is associated with severity of brain oedema following experimental subarachnoid haemorrhage in rats. J. Int. Med. Res..

[20] Arendt B.K., Walters D.K., Wu X., Tschumper R.C., Jelinek D.F. (2014). Multiple myeloma dell-derived microvesicles are enriched in CD147 expression and enhance tumor cell proliferation. Oncotarget.

[21] Jiang J.L., Zhou Q., Yu M.K., Ho L.S., Chen Z.N., Chan H.C. (2001). The involvement of HAb18G/CD147 in regulation of store-operated calcium entry and metastasis of human hepatoma cells. J. Biol. Chem..

[22] Fossum S., Mallett S., Barclay A.N. (1991). The MRC OX-47 antigen is a member of the immunoglobulin superfamily with an unusual transmembrane sequence. Eur. J. Immunol..

[23] Seulberger H., Unger C.M., Risau W. (1992). HT7, Neurothelin, Basigin, gp42 and OX-47—Many names for one developmentally regulated immuno-globulin-like surface glycoprotein on blood–brain barrier endothelium, epithelial tissue barriers and neurons. Neurosci. Lett..

[24] Liang L., Major T., Bocan T. (2002). Characterization of the promoter of human extracellular matrix metalloproteinase inducer (EMMPRIN). Gene.

[25] Kaname T., Miyauchi T., Kuwano A., Matsuda Y., Muramatsu T., Kajii T. (1993). Mapping basigin (BSG), a member of the immunoglobulin superfamily, to 19p13.3. Cytogenet. Cell Genet..

[26] Guo H., Majmudar G., Jensen T.C., Biswas C., Toole B.P., Gordon M.K. (1998). Characterization of the gene for human EMMPRIN, a tumor cell surface inducer of matrix metalloproteinases. Gene.

[27] Yu X.L., Hu T., Du J.M., Ding J.P., Yang X.M., Zhang J., Yang B., Shen X., Zhang Z., Zhong W.D. (2008). Crystal structure of HAb18G/CD147: Implications for immunoglobulin superfamily homophilic adhesion. J. Biol. Chem..

[28] Tang J., Wu Y.M., Zhao P., Yang X.M., Jiang J.L., Chen Z.N. (2008). Overexpression of HAb18G/CD147 promotes invasion and metastasis via α3β1 integrin mediated FAK–paxillin and FAK–PI3K–Ca^2+^ pathways. Cell Mol. Life Sci..

[29] Dai J.Y., Dou K.F., Wang C.H., Zhao P., Lau W.B., Tao L., Wu Y.M., Tang J., Jiang J.L., Chen Z.N. (2009). The interaction of HAb18G/CD147 with integrin α6β1 and its implications for the invasion potential of human hepatoma cells. BMC Cancer.

[30] Zhu Y., Wu J., Yuan S.Y. (2013). MCT1 and MCT4 expression during myocardial ischemic-reperfusion injury in the isolated rat heart. Cell. Physiol. Biochem..

[31] Sienel W., Polze B., Elshawi K., Lindner M., Morresi-Hauf A., Vay C., Eder F., Passlick B., Klein C.A. (2008). Cellular localization of EMMPRIN predicts prognosis of patients with operable lung adenocarcinoma independent from MMP-2 and MMP-9. Mod. Pathol..

[32] Biegler B., Kasinrerk W. (2012). Reduction of CD147 surface expression on primary T cells leads to enhanced cell proliferation. Asian Pac. J. Allergy Immunol..

[33] Caudroy S., Polette M., Nawrocki-Raby B., Cao J., Toole B.P., Zucker S., Birembaut P. (2002). EMMPRIN-mediated MMP regulation in tumor and endothelial cells. Clin. Exp. Metastasis.

[34] Jia L., Wang S., Zhou H., Cao J., Hu Y., Zhang J. (2006). Caveolin-1 up-regulates CD147 glycosylation and the invasive capability of murine hepatocarcinoma cell lines. Int. J. Biochem. Cell Biol..

[35] Ellis S.M., Nabeshima K., Biswas C. (1989). Monoclonal antibody preparation and purification of a tumor cell collagenase-stimulatory factor. Cancer Res..

[36] Cui H.Y., Guo T., Wang S.J., Zhao P., Dong Z.S., Zhang Y., Jiang J.L., Chen Z.N., Yu X.L. (2012). Dimerization is essential for HAb18G/CD147 promoting tumor invasion via MAPK pathway. Biochem. Biophys. Res. Commun..

[37] Hanna S.M., Kirk P., Holt O.J., Puklavec M.J., Brown M.H., Barclay A.N. (2003). A novel form of the membrane protein CD147 that contains an extra Ig-like domain and interacts homophilically. BMC Biochem..

[38] Redzic J.S., Armstrong G.S., Isern N.G., Jones D.N., Kieft J.S., Eisenmesser E.Z. (2011). The retinal specific CD147 Ig0 domain: From molecular structure to biological activity. J. Mol. Biol..

[39] Schlegel J., Redzic J.S., Porter C.C., Yurchenko V., Bukrinsky M., Labeikovsky W., Armstrong G.S., Zhang F., Isern N.G., DeGregori J. (2009). Solution characterization of the extracellular region of CD147 and its interaction with its enzyme ligand cyclophilin A. J. Mol. Biol..

[40] Liu T., Zhai H., Xu Y., Dong Y., Sun Y., Zang X., Zhao J. (2012). Amniotic membrane traps and induces apoptosis of inflammatory cells in ocular surface chemical burn. Mol. Vis..

[41] Taylor P.M., Woodfield R.J., Hodgkin M.N., Pettitt T.R., Martin A., Kerr D.J., Wakelam M.J. (2002). Breast cancer cell-derived EMMPRIN stimulates fibroblast MMP2 release through a phospholipase A(2) and 5-lipoxygenase catalyzed pathway. Oncogene.

[42] Maatta M., Tervahartiala T., Kaarniranta K., Tang Y., Yan L., Tuukkanen J., Sorsa T. (2006). Immunolocalization of EMMPRIN (CD147) in the human eye and detection of soluble form of EMMPRIN in ocular fluids. Curr. Eye Res..

[43] Adithi M., Nalini V., Kandalam M., Krishnakumar S. (2007). Expression of matrix metalloproteinases and their inhibitors in retinoblastoma. J. Pediatr. Hematol. Oncol..

[44] Huang Z., Tan N., Guo W., Wang L., Li H., Zhang T., Liu X., Xu Q., Li J., Guo Z. (2014). Overexpression of EMMPRIN isoform 2 is associated with head and neck cancer metastasis. PLoS One.

[45] Li H.M., Zhu P., Fan C.M., Wang Y.H., Zheng Z.H., Li X.Y., Lu N. (2006). Effects of PMA on CD147 expression in cultured THP-1 cells and monocytes of RA patients. Xi Bao Yu Fen Zi Mian Yi Xue Za Zhi.

[46] Hanata K., Yamaguchi N., Yoshikawa K., Mezaki Y., Miura M., Suzuki S., Senoo H., Ishikawa K. (2007). Soluble EMMPRIN (extra-cellular matrix metalloproteinase inducer) stimulates the migration of HEp-2 human laryngeal carcinoma cells, accompanied by increased MMP-2 production in fibroblasts. Arch. Histol. Cytol..

[47] Yang S.H., Li Y.T., Du D.Y. (2013). Oxidized low-density lipoprotein-induced CD147 expression and its inhibition by high-density lipoprotein on platelets *in vitro*. Thromb. Res..

[48] Papoutsi M., Kurz H., Schachtele C., Marme D., Christ B., Prols F., Wilting J. (2000). Induction of the blood–brain barrier marker neurothelin/HT7 in endothelial cells by a variety of tumors in chick embryos. Histochem. Cell Biol..

[49] Ran X.Y., Huang J., Zhang H.Z., Jiang Z.M., Chen J. (2013). Factors related to biologic behavior in giant cell tumor of bone. Zhonghua Bing Li Xue Za Zhi.

[50] Zhu P., Lu N., Shi Z.G., Zhou J., Wu Z.B., Yang Y., Ding J., Chen Z.N. (2006). CD147 overexpression on synoviocytes in rheumatoid arthritis enhances matrix metalloproteinase production and invasiveness of synoviocytes. Arthritis Res. Ther..

[51] Kang M.J., Kim H.P., Lee K.S., Yoo Y.D., Kwon Y.T., Kim K.M., Kim T.Y., Yi E.C. (2013). Proteomic analysis reveals that CD147/EMMPRIN confers chemoresistance in cancer stem cell-like cells. Proteomics.

[52] Walters D.K., Arendt B.K., Jelinek D.F. (2013). CD147 regulates the expression of MCT1 and lactate export in multiple myeloma cells. Cell Cycle.

[53] Chen H., Wang L., Beretov J., Hao J., Xiao W., Li Y. (2010). Co-expression of CD147/EMMPRIN with monocarboxylate transporters and multiple drug resistance proteins is associated with epithelial ovarian cancer progression. Clin. Exp. Metastasis.

[54] Zhang C., Man D.P., Ma S.M., Cao S.W., Li D.W. (2012). Expressions and significances of CD147, OPN and MMP-2 in oral squamous cell carcinoma. Sichuan Da Xue Xue Bao Yi Xue Bao.

[55] Pinheiro C., Penna V., Morais-Santos F., Abrahão-Machado L.F., Ribeiro G., Curcelli E.C., Olivieri M.V., Morini S., Valença I., Ribeiro D. (2014). Characterization of monocarboxylate transporters (MCTs) expression in soft tissue sarcomas: Distinct prognostic impact of MCT1 sub-cellular localization. J. Transl. Med..

[56] Riethdorf S., Reimers N., Assmann V., Kornfeld J.W., Terracciano L., Sauter G., Pantel K. (2006). High incidence of EMMPRIN expression in human tumors. Int. J. Cancer.

[57] Tian L., Zhang Y., Chen Y., Cai M., Dong H., Xiong L. (2013). EMMPRIN is an independent negative prognostic factor for patients with astrocytic glioma. PLoS One.

[58] Yang J.M., O’Neill P., Jin W., Foty R., Medina D.J., Xu Z., Lomas M., Arndt G.M., Tang Y., Nakada M. (2006). Extracellular matrix metalloproteinase inducer (CD147) confers resistance of breast cancer cells to Anoikis through inhibition of Bim. J. Biol. Chem..

[59] Wang W.J., Li Q.Q., Xu J.D., Cao X.X., Li H.X., Tang F., Chen Q., Yang J.M., Xu Z.D., Liu X.P. (2008). Interaction between CD147 and P-glycoprotein and their regulation by ubiquitination in breast cancer cells. Chemotherapy.

[60] Grass G.D., Tolliver L.B., Bratoeva M., Toole B.P. (2013). CD147, CD44, and the epidermal growth factor receptor (EGFR) signaling pathway cooperate to regulate breast epithelial cell invasiveness. J. Biol. Chem..

[61] Ju X.Z., Yang J.M., Zhou X.Y., Li Z.T., Wu X.H. (2008). EMMPRIN expression as a prognostic factor in radiotherapy of cervical cancer. Clin. Cancer Res..

[62] Pinheiro C., Longatto-Filho A., Pereira S.M., Etlinger D., Moreira M.A., Jube L.F., Queiroz G.S., Schmitt F., Baltazar F. (2009). Monocarboxylate transporters 1 and 4 are associated with CD147 in cervical carcinoma. Dis. Markers.

[63] Zhang F., Zeng Y.L., Zhang X.G., Chen W.J., Yang R., Li S.J. (2013). RNA interference targeting extracellular matrix metalloproteinase inducer (CD147) inhibits growth and increases chemosensitivity in human cervical cancer cells. Eur. J. Gynaecol. Oncol..

[64] Abraham D., Zins K., Sioud M., Lucas T., Aharinejad S. (2008). Host CD147 blockade by small interfering RNAs suppresses growth of human colon cancer xenografts. Front. Biosci..

[65] Nakamura K., Kodama J., Hongo A., Hiramatsu Y. (2012). Role of emmprin in endometrial cancer. BMC Cancer.

[66] Rosenthal E.L., Zhang W., Talbert M., Raisch K.P., Peters G.E. (2005). Extracellular matrix metalloprotease inducer-expressing head and neck squamous cell carcinoma cells promote fibroblast-mediated type I collagen degradation *in vitro*. Mol. Cancer Res..

[67] Liu Z., Hartman Y.E., Warram J.M., Knowles J.A., Sweeny L., Zhou T., Rosenthal E.L. (2011). Fibroblast growth factor receptor mediates fibroblast-dependent growth in EMMPRIN-depleted head and neck cancer tumor cells. Mol. Cancer Res..

[68] Sweeny L., Liu Z., Bush B.D., Hartman Y., Zhou T., Rosenthal E.L. (2012). CD147 and AGR2 expression promote cellular proliferation and metastasis of head and neck squamous cell carcinoma. Exp. Cell Res..

[69] Knowles J.A., Heath C.H., Saini R., Umphrey H., Warram J., Hoyt K., Rosenthal E.L. (2012). Molecular targeting of ultrasonographic contrast agent for detection of head and neck squamous cell carcinoma. Arch. Otolaryngol. Head Neck Surg..

[70] Sweeny L., Hartman Y.E., Zinn K.R., Prudent J.R., Marshall D.J., Shekhani M.S., Rosenthal E.L. (2013). A novel extracellular drug conjugate significantly inhibits head and neck squamous cell carcinoma. Oral Oncol..

[71] Qin Z., Dai L., Bratoeva M., Slomiany M.G., Toole B.P., Parsons C. (2011). Cooperative roles for emmprin and LYVE-1 in the regulation of chemoresistance for primary effusion lymphoma. Leukemia.

[72] Mamori S., Nagatsuma K., Matsuura T., Ohkawa K., Hano H., Fukunaga M., Matsushima M., Masui Y., Fushiya N., Onoda H. (2007). Useful detection of CD147 (EMMPRIN) for pathological diagnosis of early hepatocellular carcinoma in needle biopsy samples. World J. Gastroenterol..

[73] Kong L.M., Liao C.G., Chen L., Yang H.S., Zhang S.H., Zhang Z., Bian H.J., Xing J.L., Chen Z.N. (2011). Promoter hypomethylation up-regulates CD147 expression through increasing Sp1 binding and associates with poor prognosis in human hepatocellular carcinoma. J. Cell. Mol. Med..

[74] Fan J., Wang S., Yu S., He J., Zheng W., Zhang J. (2012). *N*-Acetylglucosaminyltransferase IVa regulates metastatic potential of mouse hepatocarcinoma cells through glycosylation of CD147. Glycoconj. J..

[75] Ke X., Li L., Dong H.L., Chen Z.N. (2012). Acquisition of anoikis resistance through CD147 upregulation: A new mechanism underlying metastasis of hepatocellular carcinoma cells. Oncol. Lett..

[76] Feng L., Zhu S., Zhang Y., Li Y., Gong L., Lan M., Han X., Yao L., Zhang W. (2013). Expression and clinical significance of HAb18G/CD147 in malignant tumors. Xi Bao Yu Fen Zi Mian Yi Xue Za Zhi.

[77] Zhang W., Zhao P., Xu X.L., Cai L., Song Z.S., Cao D.Y., Tao K.S., Zhou W.P., Chen Z.N., Dou K.F. (2013). Annexin A2 promotes the migration and invasion of human hepatocellular carcinoma cells *in vitro* by regulating the shedding of CD147-harboring microvesicles from tumor cells. PLoS One.

[78] Zhu S., Li Y., Zhang Y., Wang X., Gong L., Han X., Yao L., Lan M., Zhang W. (2014). Expression and clinical implications of HAb18G/CD147 in hepatocellular carcinoma. Hepatol. Res..

[79] Kong L.M., Liao C.G., Fei F., Guo X., Xing J.L., Chen Z.N. (2010). Transcription factor Sp1 regulates expression of cancer-associated molecule CD147 in human lung cancer. Cancer Sci..

[80] Tsai J.R., Wang H.M., Liu P.L., Chen Y.H., Yang M.C., Chou S.H., Cheng Y.J., Yin W.H., Hwang J.J., Chong I.W. (2012). High expression of heme oxygenase-1 is associated with tumor invasiveness and poor clinical outcome in non-small cell lung cancer patients. Cell. Oncol..

[81] Xu X.Y., Lin N., Li Y.M., Zhi C., Shen H. (2013). Expression of HAb18G/CD147 and its localization correlate with the progression and poor prognosis of non-small cell lung cancer. Pathol. Res. Pract..

[82] Long T., Su J., Tang W., Luo Z., Liu S., Liu Z., Zhou H., Qi M., Zeng W., Zhang J. (2013). A novel interaction between calcium-modulating cyclophilin ligand and Basigin regulates calcium signaling and matrix metalloproteinase activities in human melanoma cells. Cancer Lett..

[83] Zeng W., Su J., Wu L., Yang D., Long T., Li D., Kuang Y., Li J., Qi M., Zhang J. (2014). CD147 promotes melanoma progression through hypoxia-induced MMP2 activation. Curr. Mol. Med..

[84] Huang C.F., Zhang L., Ma S.R., Zhao Z.L., Wang W.M., He K.F., Zhao Y.F., Zhang W.F., Liu B., Sun Z.J. (2013). Clinical significance of Keap1 and Nrf2 in oral squamous cell carcinoma. PLoS One.

[85] Richard V., Sebastian P., Nair M.G., Nair S.N., Malieckal T.T., Santhosh Kumar T.R., Pillai M.R. (2013). Multiple drug resistant, tumorigenic stem-like cells in oral cancer. Cancer Lett..

[86] Siu A., Chang J., Lee C., Lee S., Lee C., Ramos D.M. (2013). Expression of EMMPRIN modulates mediators of tumor invasion in oral squamous cell carcinoma. J. Calif. Dent. Assoc..

[87] Millimaggi D., Mari M., D’Ascenzo S., Carosa E., Jannini E.A., Zucker S., Carta G., Pavan A., Dolo V. (2007). Tumor vesicle-associated CD147 modulates the angiogenic capability of endothelial cells. Neoplasia.

[88] Yang H., Chen B. (2013). CD147 in ovarian and other cancers. Int. J. Gynecol. Cancer.

[89] Zhao Y., Chen S., Gou W.F., Niu Z.F., Zhao S., Xiao L.J., Takano Y., Zheng H.C. (2013). The role of EMMPRIN expression in ovarian epithelial carcinomas. Cell Cycle.

[90] Li L., Tang W., Wu X., Karnak D., Meng X., Thompson R., Hao X., Li Y., Qiao X.T., Lin J. (2013). HAb18G/CD147 promotes pSTAT3-mediated pancreatic cancer development via CD44s. Clin. Cancer Res..

[91] Sugyo A., Tsuji A.B., Sudo H., Nagatsu K., Koizumi M., Ukai Y., Kurosawa G., Zhang M.R., Kurosawa Y., Saga T. (2013). Evaluation of (89)Zr-labeled human anti-CD147 monoclonal antibody as a positron emission tomography probe in a mouse model of pancreatic cancer. PLoS One.

[92] Wittschieber D., Stenzinger A., Klauschen F., Stephan C., Jung K., Erbersdobler A., Rabien A. (2011). Decreased RECK and Increased EMMPRIN expression in urothelial carcinoma of the bladder are associated with tumor aggressiveness. Pathobiology.

[93] Xue Y.J., Lu Q., Sun Z.X. (2011). CD147 overexpression is a prognostic factor and a potential therapeutic target in bladder cancer. Med. Oncol..

[94] Bhagirath D., Abrol N., Khan R., Sharma M., Seth A., Sharma A. (2012). Expression of CD147, BIGH3 and Stathmin and their potential role as diagnostic marker in patients with urothelial carcinoma of the bladder. Clin. Chim. Acta.

[95] Choi J.W., Kim Y., Lee J.H., Kim Y.S. (2014). Prognostic significance of lactate/proton symporters MCT1, MCT4, and their chaperone CD147 expressions in urothelial carcinoma of the bladder. Urology.

[96] Shou Z.X., Jin X., Zhao Z.S. (2012). Upregulated expression of ADAM17 is a prognostic marker for patients with gastric cancer. Ann. Surg..

[97] Chen L., Pan Y., Gu L., Nie Z., He B., Song G., Li R., Xu Y., Gao T., Wang S. (2013). ERK1/2 signalling pathway is involved in CD147-mediated gastric cancer cell line SGC7901 proliferation and invasion. Exp. Biol. Med..

[98] Yang M., Yuan Y., Zhang H., Yan M., Wang S., Feng F., Ji P., Li Y., Li B., Gao G. (2013). Prognostic significance of CD147 in patients with glioblastoma. J. Neurooncol..

[99] Gabison E.E., Mourah S., Steinfels E., Yan L., Hoang-Xuan T., Watsky M.A., de Wever B., Calvo F., Mauviel A., Menashi S. (2005). Differential expression of extracellular matrix metalloproteinase inducer (CD147) in normal and ulcerated corneas: Role in epithelio–stromal interactions and matrix metalloproteinase induction. Am. J. Pathol..

[100] Wysocki P.J. (2010). Targeted therapy of hepatocellular cancer. Expert Opin. Investig. Drugs.

[101] Xu J., Xu H.Y., Zhang Q., Song F., Jiang J.L., Yang X.M., Mi L., Wen N., Tian R., Wang L. (2007). HAb18G/CD147 functions in invasion and metastasis of hepatocellular carcinoma. Mol. Cancer Res..

[102] Li H.Y., Cao L.M. (2012). Inhibitory effect of arsenic trioxide on invasion in human hepatocellular carcinoma SMMC-7721 cells and its mechanism. Xi Bao Yu Fen Zi Mian Yi Xue Za Zhi.

[103] Gou X., Ru Q., Zhang H., Chen Y., Li L., Yang H., Xing J., Chen Z. (2009). HAb18G/CD147 inhibits starvation-induced autophagy in human hepatoma cell SMMC7721 with an involvement of Beclin 1 down-regulation. Cancer Sci..

[104] Gou X., Chen H., Jin F., Wu W., Li Y., Long J., Gong X., Luo M., Bi T., Li Z. (2014). Expressions of CD147, MMP-2 and MMP-9 in laryngeal carcinoma and its correlation with poor prognosis. Pathol. Oncol. Res..

[105] Xu X., Liu S., Lei B., Li W., Lin N., Sheng W., Huang A., Shen H. (2013). Expression of HAb18G in non-small lung cancer and characterization of activation, migration, proliferation, and apoptosis in A549 cells following siRNA-induced downregulation of HAb18G. Mol. Cell. Biochem..

[106] Ayva S.K., Karabulut A.A., Akatli A.N., Atasoy P., Bozdogan O. (2013). Epithelial expression of extracellular matrix metalloproteinase inducer/CD147 and matrix metalloproteinase-2 in neoplasms and precursor lesions derived from cutaneous squamous cells: An immunohistochemical study. Pathol. Res. Pract..

[107] Yang S., Tang C., Wang S., Song S., Liu X. (2012). Construction of a CD147 lentiviral expression vector and establishment of its stably transfected A549 cell line. Zhongguo Fei Ai Za Zhi.

[108] Szubert S., Szpurek D., Moszynski R., Nowicki M., Frankowski A., Sajdak S., Michalak S. (2014). Extracellular matrix metalloproteinase inducer (EMMPRIN) expression correlates positively with active angiogenesis and negatively with basic fibroblast growth factor expression in epithelial ovarian cancer. J. Cancer Res. Clin. Oncol..

[109] Milia-Argeiti E., Mourah S., Vallée B., Huet E., Karamanos N.K., Theocharis A.D., Menashi S. (2014). EMMPRIN/CD147-encriched membrane vesicles released from malignant human testicular germ cells increase MMP production through tumor-stroma interaction. Biochim. Biophys. Acta.

[110] Li J., Li D., Zhang L., Huang P., Li Z. (2014). Effects of *CD147* gene silencing on protein expression of ANXA2,MMP-2 and TIMP-2 by thyroid medullary carcinoma TT cells and biologic characteristics. Zhonghua Bing Li Xue Za Zhi.

[111] Dai L., Bai L., Lu Y., Xu Z., Reiss K., del Valle L., Kaleeba J., Toole B.P., Parsons C., Qin Z. (2013). Emmprin and KSHV: New partners in viral cancer pathogenesis. Cancer Lett..

[112] Dai L., Qin Z., Defee M., Toole B.P., Kirkwood K.L., Parsons C. (2012). Kaposi sarcoma-associated herpesvirus (KSHV) induces a functional tumor-associated phenotype for oral fibroblasts. Cancer Lett..

[113] Qin Z., Dai L., Slomiany M.G., Toole B.P., Parsons C. (2010). Direct activation of emmprin and associated pathogenesis by an oncogenic herpesvirus. Cancer Res..

[114] Dai L., Bratoeva M., Toole B.P., Qin Z., Parsons C. (2012). KSHV activation of VEGF secretion and invasion for endothelial cells is mediated through viral upregulation of emmprin-induced signal transduction. Int. J. Cancer.

[115] Hao J., Madigan M.C., Khatri A., Power C.A., Hung T.T., Beretov J., Chang L., Xiao W., Cozzi P.J., Graham P.H. (2012). *In vitro* and *in vivo* prostate cancer metastasis and chemoresistance can be modulated by expression of either CD44 or CD147. PLoS One.

[116] Yang Q., Liu Y., Huang Y., Huang D., Li Y., Wu J., Duan M. (2013). Expression of COX-2, CD44v6 and CD147 and relationship with invasion and lymph node metastasis in hypopharyngeal squamous cell carcinoma. PLoS One.

[117] Grass G.D., Bratoeva M., Toole B.P. (2012). Regulation of invadopodia formation and activity by CD147. J. Cell Sci..

[118] Spring F.A., Holmes C.H., Simpson K.L., Mawby W.J., Mattes M.J., Okubo Y., Parsons S.F. (1997). The Oka blood group antigen is a marker for the M6 leukocyte activation antigen, the human homolog of OX-47 antigen, basigin and neurothelin, an immunoglobulin superfamily molecule that is widely expressed in human cells and tissues. Eur. J. Immunol..

[119] Joghetaei N., Stein A., Byrne R.A., Schulz C., King L., May A.E., Schmidt R. (2013). The extracellular matrix metalloproteinase inducer (EMMPRIN, CD147)—A potential novel target in atherothrombosis prevention?. Thromb. Res..

[120] Seizer P., Gawaz M., May A.E. (2014). Cyclophilin A and EMMPRIN (CD147) in cardiovascular diseases. Cardiovasc. Res..

[121] Kanyenda L.J., Verdile G., Martins R., Meloni B.P., Chieng J., Mastaglia F., Laws S.M., Anderton R.S., Boulos S. (2014). Is cholesterol and amyloid-beta stress induced CD147 expression a protective response? Evidence that extracellular cyclophilin a mediated neuroprotection is reliant on CD147. J. Alzheimers Dis..

[122] Agrawal S.M., Williamson J., Sharma R., Kebir H., Patel K., Prat A., Yong V.W. (2013). Extracellular matrix metalloproteinase inducer shows active perivascular cuffs in multiple sclerosis. Brain.

[123] Zhang D.W., Zhao Y.X., Wei D., Li Y.L., Zhang Y., Wu J., Xu J., Chen C., Tang H., Zhang W. (2012). HAb18G/CD147 promotes activation of hepatic stellate cells and is a target for antibody therapy of liver fibrosis. J. Hepatol..

[124] Zhao S., Chen C., Liu S., Zeng W., Su J., Wu L., Luo Z., Zhou S., Li Q., Zhang J. (2013). CD147 promotes MTX resistance by immune cells through up-regulating ABCG2 expression and function. J. Dermatol. Sci..

[125] Lee C.L., Lam M.P., Lam K.K., Leung C.O., Pang R.T., Chu I.K., Wan T.H., Chai J., Yeung W.S., Chiu P.C. (2013). Identification of CD147 (basigin) as a mediator of trophoblast functions. Hum. Reprod..

[126] Richard V., Pillai M.R. (2010). The stem cell code in oral epithelial tumorigenesis: “The cancer stem cell shift hypothesis”. Biochim. Biophys. Acta.

[127] Jin A., Chen H., Wang C., Tsang L.L., Jiang X., Cai Z., Chan H.C., Zhou X. (2014). Elevated expression of CD147 in patients with endometriosis and its role in regulating apoptosis and migration of human endometrial cells. Fertil. Steril..

[128] Pistol G., Matache C., Calugaru A., Stavaru C., Tanaseanu S., Ionescu R., Dumitrache S., Stefanescu M. (2007). Roles of CD147 on T lymphocytes activation and MMP-9 secretion in systemic lupus erythematosus. J. Cell. Mol. Med..

[129] Wang Z.Z., Wang Y., Li J.M., Mou F.X., Wu H. (2013). Significance of serum MMP-3, TIMP-1, and monocyte CD147 in rheumatoid arthritis patients of damp-heat Bi-syndrome and of cold-damp Bi-syndrome. Zhongguo Zhong Xi Yi Jie He Za Zhi.

[130] Maeda-Hori M., Kosugi T., Kojima H., Sato W., Inaba S., Maeda K., Nagaya H., Sato Y., Ishimoto T., Ozaki T. (2014). Plasma CD147 reflects histological features in patients with lupus nephritis. Lupus.

[131] Wang M., Huang Z.X., Pan Y.F., Zhang F.C., Zheng B.R., Deng W.M., Li T.W., Gu J.R. (2010). Expressions of CD147 in peripheral monocytes and T lymphocytes of patients with ankylosing spondylitis. Zhonghua Yi Xue Za Zhi.

[132] Agrawal S.M., Silva C., Tourtellotte W.W., Yong V.W. (2011). EMMPRIN: A novel regulator of leukocyte transmigration into the CNS in multiple sclerosis and experimental autoimmune encephalomyelitis. J. Neurosci..

[133] Wang B., Xu S.S., Jiang J.J., Lu X.B., Xue Y.S., Wang J.C., Mi Y.F., Zhu M., Ge W.L., Tang L.J. (2012). Expression of extracellular matrix metalloproteinase inducer in the unstable plaque of patients with acute coronary syndrome. Zhonghua Xin Xue Guan Bing Za Zhi.

[134] Zhou J., Song B., Duan X., Long Y., Lu J., Li Z., Zeng S., Zhan Q., Yuan M., Yang Q. (2014). Association of BSG genetic polymorphisms with atherosclerotic cerebral infarction in the Han Chinese population. Int. J. Neurosci..

[135] Nishioku T., Dohgu S., Koga M., Machida T., Watanabe T., Miura T., Tsumagari K., Terasawa M., Yamauchi A., Kataoka Y. (2012). Cyclophilin A secreted from fibroblast-like synoviocytes is involved in the induction of CD147 expression in macrophages of mice with collagen-induced arthritis. J. Inflamm..

[136] Geng J.J., Zhang K., Chen L.N., Miao J.L., Yao M., Ren Y., Fu Z.G., Chen Z.N., Zhu P. (2014). Enhancement of CD147 on M1 macrophages induces differentiation of Th17 cells in the lung interstitial fibrosis. Biochim. Biophys. Acta.

[137] Philp N.J., Ochrietor J.D., Rudoy C., Muramatsu T., Linser P.J. (2003). Loss of MCT1, MCT3, and MCT4 expression in the retinal pigment epithelium and neural retina of the 5A11/basigin-null mouse. Investig. Ophthalmol. Vis. Sci..

[138] Payne K.J., Clyde L.A., Weldon A.J., Milford T.A., Yellon S.M. (2012). Residency and activation of myeloid cells during remodeling of the prepartum murine cervix. Biol. Reprod..

[139] Crosnier C., Bustamante L.Y., Bartholdson S.J., Bei A.K., Theron M., Uchikawa M., Mboup S., Ndir O., Kwiatkowski D.P., Duraisingh M.T. (2011). Basigin is a receptor essential for erythrocyte invasion by *Plasmodium falciparum*. Nature.

[140] Williams A.R., Douglas A.D., Miura K., Illingworth J.J., Choudhary P., Murungi L.M., Furze J.M., Diouf A., Miotto O., Crosnier C. (2012). Enhancing blockade of *Plasmodium falciparum* erythrocyte invasion: Assessing combinations of antibodies against PfRH5 and other merozoite antigens. PLoS Pathog..

[141] Muramatsu T. (2012). Basigin: A multifunctional membrane protein with an emerging role in infections by malaria parasites. Expert Opin. Ther. Targets.

[142] Wanaguru M., Liu W., Hahn B.H., Rayner J.C., Wright G.J. (2013). RH5-Basigin interaction plays a major role in the host tropism of *Plasmodium falciparum*. Proc. Natl. Acad. Sci. USA.

[143] Bernard S.C., Simpson N., Join-Lambert O., Federici C., Laran-Chich M.P., Maïssa N., Bouzinba-Ségard H., Morand P.C., Chretien F., Taouji S. (2014). Pathogenic *Neisseria meningitidis* utilizes CD147 for vascular colonization. Nat. Med..

[144] Bartholdson S.J., Crosnier C., Bustamante L.Y., Rayner J.C., Wright G.J. (2013). Identifying novel *Plasmodium falciparum* erythrocyte invasion receptors using systematic extracellular protein interaction screens. Cell. Microbiol..

[145] Zhou S., Liao L., Chen C., Zeng W., Liu S., Su J., Zhao S., Chen M., Kuang Y., Chen X. (2013). CD147 mediates chemoresistance in breast cancer via ABCG2 by affecting its cellular localization and dimerization. Cancer Lett..

[146] Slomiany M.G., Grass G.D., Robertson A.D., Yang X.Y., Maria B.L., Beeson C., Toole B.P. (2009). Hyaluronan, CD44, and emmprin regulate lactate efflux and membrane localization of monocarboxylate transporters in human breast carcinoma cells. Cancer. Res..

[147] Toole B.P., Slomiany M.G. (2008). Hyaluronan, CD44 and Emmprin: Partners in cancer cell chemoresistance. Drug Resist. Updates.

[148] Huang Z., Wang L., Wang Y., Zhuo Y., Li H., Chen J., Chen W. (2013). Overexpression of CD147 contributes to the chemoresistance of head and neck squamous cell carcinoma cells. J. Oral Pathol. Med..

[149] Zhang X., Liu P., Zhang B., Wang A., Yang M. (2010). Role of STAT3 decoy oligodeoxynucleotides on cell invasion and chemosensitivity in human epithelial ovarian cancer cells. Cancer Genet. Cytogenet..

[150] Amit-Cohen B.C., Rahat M.M., Rahat M.A. (2013). Tumor cell-macrophage interactions increase angiogenesis through secretion of EMMPRIN. Front. Physiol..

[151] Singh M., Kindelberger D., Nagymanyoki Z., Ng S.W., Quick C.M., Yamamoto H., Fichorova R., Fulop V., Berkowitz R.S. (2012). Vascular endothelial growth factors and their receptors and regulators in gestational trophoblastic diseases and normal placenta. J. Reprod. Med..

[152] Mishra B., Kizaki K., Sato T., Ito A., Hashizume K. (2012). The role of extracellular matrix metalloproteinase inducer (EMMPRIN) in the regulation of bovine endometrial cell functions. Biol. Reprod..

[153] Mishra B., Kizaki K., Koshi K., Ushizawa K., Takahashi T., Hosoe M., Sato T., Ito A., Hashizume K. (2012). Expression of extracellular matrix metalloproteinase inducer (EMMPRIN) and its expected roles in the bovine endometrium during gestation. Domest. Anim. Endocrinol..

[154] Bahmed K., Henry C., Holliday M., Redzic J., Ciobanu M., Zhang F., Weekes C., Sclafani R., Degregori J., Eisenmesser E. (2012). Extracellular cyclophilin-A stimulates ERK1/2 phosphorylation in a cell-dependent manner but broadly stimulates nuclear factor kappa B. Cancer Cell Int..

[155] Schmidt R., Bultmann A., Fischel S., Gillitzer A., Cullen P., Walch A., Jost P., Ungerer M., Tolley N.D., Lindemann S. (2008). Extracellular matrix metalloproteinase inducer (CD147) is a novel receptor on platelets, activates platelets, and augments nuclear factor kappaB-dependent inflammation in monocytes. Circ. Res..

[156] Yang Y., Lu N., Zhou J., Chen Z.N., Zhu P. (2008). Cyclophilin A up-regulates MMP-9 expression and adhesion of monocytes/macrophages via CD147 signalling pathway in rheumatoid arthritis. Rheumatology.

[157] Pinheiro C., Reis R.M., Ricardo S., Longatto-Filho A., Schmitt F., Baltazar F. (2010). Expression of monocarboxylate transporters 1, 2, and 4 in human tumours and their association with CD147 and CD44. J. Biomed. Biotechnol..

[158] Klawitter J., Klawitter J., Schmitz V., Brunner N., Crunk A., Corby K., Bendrick-Peart J., Leibfritz D., Edelstein C.L., Thurman J.M. (2012). Low-salt diet and cyclosporine nephrotoxicity: Changes in kidney cell metabolism. J. Proteome Res..

[159] Hoffmann H., Schiene-Fischer C. (2014). Functional aspects of extracellular cyclophilins. Biol. Chem..

[160] Venkatesan B., Valente A.J., Prabhu S.D., Shanmugam P., Delafontaine P., Chandrasekar B. (2010). EMMPRIN activates multiple transcription factors in cardiomyocytes, and induces interleukin-18 expression via Rac1-dependent PI3K/Akt/IKK/NF-κB andMKK7/JNK/AP-1 signaling. J. Mol. Cell. Cardiol..

[161] Redzic J.S., Kendrick A.A., Bahmed K., Dahl K.D., Pearson C.G., Robinson W.A., Robinson S.E., Graner M.W., Eisenmesser E.Z. (2013). Extracellular vesicles secreted from cancer cell lines stimulate secretion of MMP-9, IL-6, TGF-β1 and EMMPRIN. PLoS One.

[162] Wu J., Ru N.Y., Zhang Y., Li Y., Wei D., Ren Z., Huang X.F., Chen Z.N., Bian H. (2011). HAb18G/CD147 promotes epithelial-mesenchymal transition through TGF-β signaling and is transcriptionally regulated by Slug. Oncogene.

[163] Menashi S., Serova M., Ma L., Vignot S., Mourah S., Calvo F. (2003). Regulation of extracellular matrix metalloproteinase inducer and matrix metalloproteinase expression by amphiregulin in transformed human breast epithelial cells. Cancer Res..

[164] Capestrano M., Mariggio S., Perinetti G., Egorova A.V., Iacobacci S., Santoro M., di Pentima A., Iurisci C., Egorov M.V., di Tullio G. (2014). Cytosolic phospholipase A_2_ε drives recycling through the clathrin-independent endocytic route. J. Cell Sci..

[165] Mauris J., Woodward A.M., Cao Z., Panjwani N., Argüeso P. (2014). Molecular basis for MMP9 induction and disruption of epithelial cell–cell contacts by galectin-3. J. Cell Sci..

[166] Le Floch R., Chiche J., Marchiq I., Naiken T., Ilc K., Murray C.M., Critchlow S.E., Roux D., Simon M.P., Pouyssegur J. (2011). CD147 subunit of lactate/H^+^ symporters MCT1 and hypoxia-inducible MCT4 is critical for energetics and growth of glycolytic tumors. Proc. Natl. Acad. Sci. USA.

[167] Priglinger C.S., Szober C.M., Priglinger S.G., Merl J., Euler K.N., Kernt M., Gondi G., Behler J., Geerlof A., Kampik A. (2013). Galectin-3 induces clustering of CD147 and integrin-β1 transmembrane glycoprotein receptors on the RPE cell surface. PLoS One.

[168] Feng X., Xiu B., Xu L., Yang X., He J., Leong D., He F., Zhang H. (2011). Hepatitis C virus core protein promotes the migration and invasion of hepatocyte via activating transcription of extracellular matrix metalloproteinase inducer. Virus Res..

[169] Reddy V.S., Prabhu S.D., Mummidi S., Valente A.J., Venkatesan B., Shanmugam P., Delafontaine P., Chandrasekar B. (2010). Interleukin-18 induces EMMPRIN expression in primary cardiomyocytes via JNK/Sp1 signaling and MMP-9 in part via EMMPRIN and through AP-1 and NF-κB activation. Am. J. Physiol. Heart Circ. Physiol..

[170] Kong L.M., Liao C.G., Zhang Y., Xu J., Li Y., Huang W., Zhang Y., Bian H., Chen Z.N. (2014). A regulatory loop involving miR-22, Sp1 and c-Myc modulates CD147 expression in breast cancer invasion and metastasis. Cancer Res..

[171] Gao J., Gao X., Pan S. (2013). Effect of minocycline on carotid atherosclerotic plaques. Neurol. Res..

[172] Tang W., Hemler M.E. (2004). Caveolin-1 regulates matrix metalloproteinases-1 induction and CD147/EMMPRIN cell surface clustering. J. Biol. Chem..

[173] Shi Y., Simmons M.N., Seki T., Oh S.P., Sugrue S.P. (2001). Change in gene expression subsequent to induction of Pnn/DRS/memA: Increase in *p21* (*cip1*/*waf1*). Oncogene.

[174] Zhang X., Tang X., Liu H., Li L., Hou Q., Gao J. (2012). Autophagy induced by baicalin involves downregulation of CD147 in SMMC-7721 cells *in vitro*. Oncol. Rep..

[175] Landskron J., Tasken K. (2013). CD147 in regulatory T cells. Cell. Immunol..

[176] Xu X.Y., Zhi C., Li Y.M., Qi W.J., Mei J.J., Yan Z.M., Shen H. (2012). Association of HAb18G with clinicopathologic features and prognosis in non-small cell carcinoma of lung. Zhonghua Bing Li Xue Za Zhi.

[177] Boye K., Nesland J.M., Sandstad B., Haugland Haugen M., Maelandsmo G.M., Flatmark K. (2012). EMMPRIN is associated with S100A4 and predicts patient outcome in colorectal cancer. Br. J. Cancer.

[178] Grupp K., Hohne T.S., Prien K., Hube-Magg C., Tsourlakis M.C., Sirma H., Pham T., Heinzer H., Graefen M., Michl U. (2013). Reduced CD147 expression is linked to ERG fusion-positive prostate cancers but lacks substantial impact on PSA recurrence in patients treated by radical prostatectomy. Exp. Mol. Pathol..

[179] Niu H., Wang R., Cheng J., Gao S., Liu B. (2013). Treatment of (131)I-labeled anti-CD147 monoclonal antibody in VX2 carcinoma-induced liver tumors. Oncol. Rep..

[180] Agrawal S.M., Silva C., Wang J., Tong J.P., Yong V.W. (2012). A novel anti-EMMPRIN function-blocking antibody reduces T cell proliferation and neurotoxicity: Relevance to multiple sclerosis. J. Neuroinflamm..

[181] Li Y., Xu J., Chen L., Zhong W.D., Zhang Z., Mi L., Zhang Y., Liao C.G., Bian H.J., Jiang J.L. (2009). HAb18G (CD147), a cancer-associated biomarker and its role in cancer detection. Histopathology.

[182] Zou W., Yang H., Hou X., Zhang W., Chen B., Xin X. (2007). Inhibition of *CD147* gene expression via RNA interference reduces tumor cell invasion, tumorigenicity and increases chemosensitivity to paclitaxel in HO-8910pm cells. Cancer Lett..

[183] Yurchenko V., Constant S., Eisenmesser E., Bukrinsky M. (2010). Cyclophilin-CD147 interactions: A new target for anti-inflammatory therapeutics. Clin. Exp. Immunol..

